# Self-organization Assay for Min Proteins of *Escherichia coli* in Micro-droplets Covered with Lipids

**DOI:** 10.21769/BioProtoc.3561

**Published:** 2020-03-20

**Authors:** Shunshi Kohyama, Kei Fujiwara, Natsuhiko Yoshinaga, Nobuhide Doi

**Affiliations:** 1 Department of Biosciences and Informatics, Keio University, Yokohama, Japan; 2 Mathematical Science Group, WPI Advanced Institute for Materials Research (WPI-AIMR), Tohoku University, Sendai, Japan; 3 MathAM-OIL, AIST, Sendai, Japan

**Keywords:** Spatiotemporal regulation, Artificial cells, Cell-sized space, Min system, Cell division, Reaction-diffusion coupling, *In vitro* reconstitution

## Abstract

The Min system determines the cell division plane of bacteria. As a cue of spatiotemporal regulation, the Min system uses wave propagation of MinD protein (Min wave). Therefore, the reconstitution of the Min wave in cell-sized closed space will lead to the creation of artificial cells capable of cell division. The Min waves emerge via coupling between the reactions among MinD, MinE, and ATP and the differences in diffusion rate on the cell membrane and in the cytoplasm. Because Min waves appear only under the balanced condition of the reaction-diffusion coupling, special attentions are needed towards several technical points for the reconstitution of Min waves in artificial cells. This protocol describes a technical method for stably generating Min waves in artificial cells.

## Background

Min system, which determines the cell center for symmetric cell division, is one of the most striking examples of intracellular organization systems in bacteria (Rothfield *et al.*, 2005; Rowlett and Margolin, 2013). Min system uses pattern formation of time-dependent protein gradient inside cells known as Min waves (Loose *et al.*, 2008; Halatek and Frey, 2012; Bonny *et al.*, 2013; Zieske *et al.*, 2016; Kohyama *et al.*, 2019). The Min wave is emerged by a reaction-diffusion coupling of two proteins: MinD and MinE. By binding to ATP, MinD forms dimer and attach to membrane. MinE is recruited to ATP-MinD and induces ATPase activity of MinD. By MinE, ATP-MinD becomes ADP-MinD, and are detached from the membrane. ADP-MinD is converted back to ATP-MinD by binding ATP in cytosol (Huang *et al.*, 2003; Halatek and Frey, 2012). This sequence of reactions continuously proceed. Molecular diffusion of MinD and MinE are different between on membrane and in cytosol. By coupling the difference of molecular diffusion and the continuous reactions, Min waves including pole-to-pole oscillation of MinDE proteins *in vivo* appears (Huang *et al.*, 2003; Halatek and Frey, 2012; Bonny *et al.*, 2013; Wu *et al.*, 2015).

 To characterize the Min wave behavior *in vitro*, its reconstitution system on 2D planar membranes has been developed (Loose *et al.*, 2008 and 2011; Zieske and Schwille, 2013; Vecchiarelli *et al.*, 2013 and 2016; Denk *et al.*, 2018). The 2D system has shown that MinD, MinE, and ATP are necessary and sufficient for the Min wave emergence. Although the 2D system has contributed to elucidation of the Min wave characteristics, our recent study showed that the condition of the Min wave emergence is drastically shifted when Min proteins (MinD and MinE) are confined in cell-sized spaces (Kohyama *et al.*, 2019; Yoshida *et al.*, 2019). Therefore, characterization of the Min wave behavior under cell-sized confinement conditions will be necessary for emulating real traits of the Min wave in living cells. In this report, we introduce the method to emerge the Min wave in a cell-sized space made by water-in-oil emulsion.

## Materials and Reagents

1. AmiconUltra-15 10k filter (Merck Millipore, catalog number: UFC901024)
2. AmiconUltra-0.5 10k filter (Merck Millipore, catalog number: UFC501096)
3. AmiconUltra-15 30k filter (Merck Millipore, catalog number: UFC903024)
4. AmiconUltra-0.5 30k filter (Merck Millipore, catalog number: UFC503096)
5. AmiconUltra-15 50k filter (Merck Millipore, catalog number: UFC905024)
6. AmiconUltra-0.5 50k filter (Merck Millipore, catalog number: UFC505096)
7. Glass microtube No.2 (Maruemu, catalog number: 310109)
8. Neo Micro cover glass 25 mm x 36 mm (Matsunami Glass, catalog number: 25 x 36 No.1)
9. Micro cover glass 18 mm x 18 mm (Matsunami Glass, catalog number: 18 x 18 No.1)
10. Double-sided Tape NICE TACK(Nichiban, NW-10)
11. 0.2 ml tube (Nippon Genetics, catalog number: FG-021F)
12. 0.6 ml tube (Axygen, catalog number: MCT-060-C)
13. 1.7 ml tube (Axygen, catalog number: MCT-175-X)
14. 15 ml centrifuge tubes (Nichiryo, catalog number: 1930907)
15. 50 ml centrifuge tubes (Nichiryo, catalog number: 1910214)
16. DuraSeal (Diversified Biotech, TestTubeSEAL, 1" x 150')
17. Polyprep chromatography column (Bio-Rad, catalog number: 731-1550)
18. cOmplete His-Tag purification resin (Roche, Basel, catalog number: 5893682001)
19. pET15-His-sfGFP-minD (constructed in Kohyama *et al.*, 2019, Note 1)
20. pET29-minE-mCherry-His (constructed in Kohyama *et al.*, 2019, Note 1)
21. Tris(hydroxymethyl)aminomethane (Nacalai Tesque, catalog number: 35434-05)
22. L-Glutamic acid potassium salt monohydrate ≥ 99% (HPLC), powder (GluK) (Sigma-Aldrich, catalog number: G1501)
23. Bovine serum albumin (BSA) (Sigma-Aldrich, catalog number: A6003)
24. HiTrap Q HP (GE Healthcare, catalog number: 17115301)
25. Adenosine-5’-triphosphate Magnesium Salt (ADP) (Nacalai Tesque, catalog number: 00386-54)
26. L-Glutamic acid hemimagnesium salt tetrahydrate (GluMg) (Sigma-Aldrich, catalog number: 49605)
27. Isopropyl-β-D(-)-thiogalactopyranoside (IPTG) (Nacalai Tesque, catalog number: 19742-94)
28. Sodium dihydrogen phosphate (NaH_2_PO_4_) (Nacalai Tesque, catalog number: 31737-65)
29. di-Sodium hydrogenphosphate (Na_2_HPO_4_) (Nacalai Tesque, catalog number: 31726-05)
30. Sodium chloride (NaCl) (Nacalai Tesque, catalog number: 31320-05)
31. Imidazole (Nacalai Tesque, catalog number: 19004-35)
32. Dithiothreitol (DTT) (Nacalai Tesque, catalog number: 14128-04)
33. Phenylmethylsulfonyl fluoride (PMSF) (Nacalai Tesque, catalog number: 27327-94)
34. HEPES (Nacalai Tesque, catalog number: 17546-05)
35. Glycerol (Nacalai Tesque, catalog number: 17018-25)
36. 0.5 mol/L EDTA solution (pH 8.0) (Nacalai Tesque, catalog number: 14347-21)
37. *Escherichia coli* BL21-CodonPlus(DE3)-RIPL (Agilent Technologies, catalog number: 230280) (Note 2)
38. Adenosine-5'-diphosphate Sodium Salt from Bacterial Source (ADP) (Nacalai Tesque, catalog number: 01652-24)
39. Sodium Dodecyl Sulfate (SDS) (Nacalai Tesque, catalog number: 31606-75)
40. Acrylamide(monomer) (Nacalai Tesque, catalog number: 00809-85)
41. N,N'-Methylenebisacrylamide (Nacalai Tesque, catalog number: 22407-52)
42. Coomassie Brilliant Blue (CBB) (Nacalai Tesque, catalog number: 09409-42)
43. Hydrochloric Acid (35%) (HCl) (Nacalai Tesque, catalog number: 18321-05)
44. Pierce BCA Protein assay kit (catalog number: 23227)
45. *E. coli* Extract Polar (Avanti, catalog number: 100600C)
46. Mineral oil (Nacalai Tesque, catalog number: 23306-84)
47. Ultrapure water (MilliQ water)
48. Argon gas
49. Ampicillin Sodium Salt (Nacalai Tesque, catalog number: 02739-32)
50. Kanamycin Sulfate (Nacalai Tesque, catalog number: 19860-44)
51. Bacto tryptone (BD, catalog number: 211705)
52. Yeast extract (BD, catalog number: 212750)
53. Agar (Nacalai Tesque, catalog number: 01028-85)
54. Potassium Hydroxide (KOH) (Nacalai Tesque, catalog number: 28616-45)
55. Citric Acid, Anhydrous (Nacalai Tesque, catalog number: 09109-85)
56. β-cyclodextrin (Wako Chemicals, catalog number: 038-08343)
57. Ethanol (Nacalai Tesque, catalog number: 14713-53)
58. LB medium (see Recipes)
59. LB agar medium with an antibiotic (see Recipes)
60. Sodium phosphate buffer (pH 7.6) (see Recipes)
61. HEPES-KOH (pH 7.6) (see Recipes)
62. Tris-HCl (pH 7.6) (see Recipes)
63. LS buffer (see Recipes)
64. LS-ADP buffer (see Recipes)
65. WS buffer (see Recipes)
66. EL buffer (see Recipes)
67. Storage buffer (see Recipes)
68. HG buffer (see Recipes)
69. IEX-A buffer (see Recipes)
70. IEX-B buffer (see Recipes)
71. CBB staining solution (see Recipes)
72. RE buffer (see Recipes)

## Equipment

1. AKTA start (GE Healthcare, catalog number: 29022094)
2. Frac30 (GE Healthcare, catalog number: 29023051)
3. Bransonic (Branson, model: CPX1800H-J)
4. Sonifier Analog Series (Branson, model: 250)
5. Centrifuge (KUBOTA, model: 5922)
6. pH meter (HORIBA, LAQUA F-52 with 9615S-10D)
7. Inverted microscope (Zeiss, AxioObserver Z1) with cMOS camera (Hamamatsu Photonics, ORCA-FLASH4.0 V2) and ScopeLED Light Sources (BioVision Technologies, F-Series 390/470/560P/640) regulated by micromanager 1.4 (R. Vale Laboratory). The filter cubes were purchased from Thorlabs (MDF-GFP for GFP tracking and MDF-CY3.5 for mCherry tracking)
8. ChemDoc Touch MP (Bio-Rad, catalog number: 17001402JA)
9. Vortex mixer (Scientific Industries, model: Vortex-Genie 2)
-30 °C refrigerator
-80 °C refrigerator
12. Autoclave

## Software

1. Fiji software (National Institutes of Health, https://fiji.sc/)

## Procedure

- Sample preparation
  1. Expression of His-sfGFP-MinD and MinE-mCherry-His
    - Transformation to introduce the expression plasmids.
    - Gently mix 100 μl of chemical competent cells of *E. coli* BL21-CodonPlus(DE3)-RIPL (Note 2) with 1-10 ng of pET15b-sfGFP-MinD or pET29-minE-mCherry-His (Note 1).
    - On ice 30 min.
    - Heat shock at 42 °C for 1 min.
    - Chilled on ice again.
    - Add 400 μl of SOB medium to the cells, and incubate at 37 °C for 30 min.
    - Plating LB agar medium with 100 μg/ml Ampicillin (for MinD) or with 25 μg/ml Kanamycin (for MinE).
    - Incubate the plate at 37 °C for 12-18 h.
    - Pick up a single colony of the plate and inoculate in 4 ml LB medium with 100 μg/ml Ampicillin (MinD) or with 25 μg/ml Kanamycin (MinE).
    - Pre-culture cells in LB medium at 37 °C for 12-18 h.
    - Transfer 2 ml of the pre-culture to 200 ml LB medium with 100 μg/ml Ampicillin (MinD) or with 25 μg/ml Kanamycin (MinE).
    - Add IPTG to 1 mM final at the time OD_600_ reaches 0.1-0.2 (MinD) or 0.6-0.7 (MinE).
    - Further cultivate cells at 37 °C, 180 rpm for 3-4 h (MinD) or at 16 °C, 120 rpm for 12 h (MinE).
    - Transfer the cultivated culture to 50 ml centrifuge tubes and collect the cells by centrifugation at 8,000 *× g* at 4 °C for 2 min.
    - Store the cells at -80 °C after removal of supernatant if the experiment should be stopped at this point.
  2. Purification of His-sfGFP-MinD
    - Suspend the stored cells expressing His-sfGFP-MinD in 4 ml LS-ADP buffer.
    - Disrupt cells by sonication using Sonifier Analog Series at duty 30%, output 3, for 30 min (Note 3).
    - Collect supernatant of the disrupted cells after centrifugation at 20,000 *× g* at 4 °C for 30 min.
    - Mix the supernatant with 500 μl of cOmplete His-Tag purification resin at 4 °C for 30 min under gently rotating.
    - Load the resin onto a polyprep chromatography column.
    - Wash the loaded resin with 25 ml WS buffer.
    - Elute the target protein with 2 ml EL buffer.
    - To exchange the EL buffer with storage buffer with 0.2 mM ADP, transfer the eluted solution to AmiconUltra-15 30k filters.
    - Add 8 ml of storage buffer.
    - Centrifuge the device at 4,000 *× g* at 4 °C. Concentrate the solution less than 2 ml (generally, 30-60 min continuous centrifugation is needed).
    - Repeat Steps A2i-A2j (total 5-6 times).
    - Transfer the concentrated solution to AmiconUltra-0.5 30k filters.
    - Centrifuge the device at 14,000 *× g* at 4 °C to concentrate the solution less than 0.1 ml (generally, it takes 30 min).
    - Estimate protein purity and concentrations by CBB staining after separating by SDS-PAGE (see quantification by CBB staining) and bicinchoninic acid (BCA) assay (according to the manufacturer’s procedure).
    - Adjust protein concentration of the purified protein at 100 μM by the storage buffer and store the protein at -80 °C before usage (Note 4).
  3. Purification of MinE-mCherry-His (Note 5)
    - Suspend the stored cells expressing MinE-mCherry-His in 4 ml LS buffer.
    - Disrupt cells by sonication using Sonifier Analog Series at duty 30% (cycles of 0.3 s ON and 0.7 s OFF), output 3 (out of 10), for 30 min (Note 3).
    - Collect supernatant of the disrupted cells after centrifugation at 20,000 *× g* at 4 °C for 30 min.
    - Mix the supernatant with 500 μl of cOmplete His-Tag purification resin at 4 °C for 30 min under gently rotating.
    - Load the resin onto a polyprep chromatography column.
    - Wash the loaded resin with 25 ml WS buffer.
    - Elute the target protein with 2 ml EL buffer.
    - Dilute the elution fraction with 5- to 10-fold HG buffer.
    - Apply the diluted solution to HiTrap Q HP column equilibrated with IEX-A buffer by using AKTA start.
    - By using an ion exchange protocol (basically installed in AKTA start as “IEX exchange”), wash the column loaded with IEX-A buffer, and elute proteins by gradient mixing of IEX-A and IEX-B buffer.
    - Determine the peak fractions of MinE-mCherry-His by SDS-PAGE, and use the peak fractions further.
    - To exchange the buffer with Storage buffer, transfer the eluted solution to AmiconUltra-15 10k filters.
    - Add 8 ml of storage buffer.
    - Centrifuge the device at 4,000 *× g* at 4 °C. Concentrate the solution less than 2 ml (generally, 30 min-60 min continuous centrifugation is needed).
    - Repeat Steps A3m-A3n (total 5-6 times).
    - Transfer the concentrated solution to AmiconUltra-0.5 10k filters.
    - Centrifuge the device at 14,000 *× g* at 4 °C to concentrate the solution less than 0.1 ml (generally, it takes 30 min).
    - Estimate protein purity and concentrations by CBB staining after separating by SDS-PAGE (see quantification by CBB staining) and BCA assay (according to the manufacturer’s procedure).
    - Adjust protein concentration of the purified protein at 100 μM by the storage buffer and store the protein at -80 °C before usage (Note 4).
  4. Washing BSA
    - Take 100~200 mg BSA in 15 ml centrifuge tubes.
    - Add the 10 ml of MQ water to the tube and dissolve BSA by vortexing.
    - Transfer the BSA solution to AmiconUltra-15 50k filters.
    - Centrifuge the device at 4,000 *× g* at 4 °C to concentrate the solution less than 2 ml (generally, it takes 30-60 min).
    - Add 8 ml of RE buffer.
    - Repeat Steps A4d-A4e (total 5-6 times).
    - Concentrate the solution less than 2 ml by centrifugation at 4,000 *× g* at 4 °C.
    - Transfer concentrated solution to AmiconUltra-0.5 50k filters.
    - Centrifuge the device at 14,000 *× g* at 4 °C for 30 min to further concentrate the solution less than 0.3 ml.
    - Estimate protein concentrations by BCA assay (according to the manufacturer’s procedure).
    - Store the BSA solution in the storage buffer at RT before usage (typically 300-400 mg/ml. BSA solution should renew once in a month.
  5. Protein quantification by CBB staining
    - Load samples and dilution series of BSA (200, 400, 600, 800, 1,000 ng) on 12.5% SDS-PAGE gels (by 12.5% or 15% acrylamide with 1/37.5 of N,N'-Methylenebisacrylamide for His-sfGFP-MinD or MinE-mCherry-His, respectively).
    - Run SDS-PAGE (typically, 20 mA, 60 min).
    - Stain the gels by CBB solution at room temperature for 30 min.
    - Remove redundant dye by immersing the gels in water at room temperature for 30 min
    - Take the image of CBB stained gels by ChemDoc touch MP.
    - Measure band intensities of BSA by Fiji software to construct a standard curve (Note 6).
    - Quantify the amount of the sample loaded by measuring band intensity and using the standard curve (Note 6).
    - Convert the amount to the concentration of proteins.
- Self-organization assay inside lipid droplets
  1. Preparation of lipid oil
    - The general protocol for microdroplets preparation was followed according to a previous report (Fujiwara and Yanagisawa, 2014). Add 20 µl of 25 mg/ml *E. coli* polar lipid extract (dissolved in chloroform and stored at -30 °C, total 1 mg) into a round glass microtube (Maruemu Glass microtube No.2) (Figure 1A, Note 7).
    - Dry up the lipid solution under gentle argon gas flow (Figures 1B-1D, Notes 7-9).
    - Add 1 ml mineral oil to the dried lipid film to be 1 mg/ml (Note 7).
    - Seal the glass tubes by DuraSeal.
    - Sonicate the lipid tube to dissolve lipids with the mineral oil for 90 min at 60 °C by using Bransonic.
    - Vortex the sonicated lipid oil for 1 min at room temperature.
    - Store the lipid oil at room temperature and must use in the day. Do not chill on ice or storage in refrigerators.
  2. Microdroplets preparation
    - Make the reaction mixture consisted of 1 µM (0.58 mg/ml) His-sfGFP-MinD, 1 µM (0.38 mg/ml) MinE-mCherry-His, 2.5 mM ATP, 100 mg/ml washed BSA in RE buffer in a 0.2 ml tube.
    - Add 100 μl of lipid oil into a 0.6 ml tube and pour 2 μl of the reaction mixture to the lipid oil (Figure 2A).
    - Gently tapping the tube about 10 times for emulsification (Figure 2B).
    **Figure 1.**
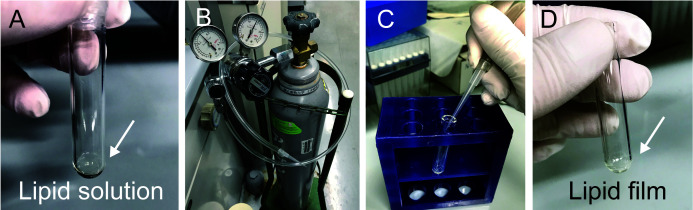
Preparation of lipid films. A. Lipid solution in a round glass microtube. B. Argas with a regulator. C. Drying step. D. Lipid films.
    **Figure 2.**
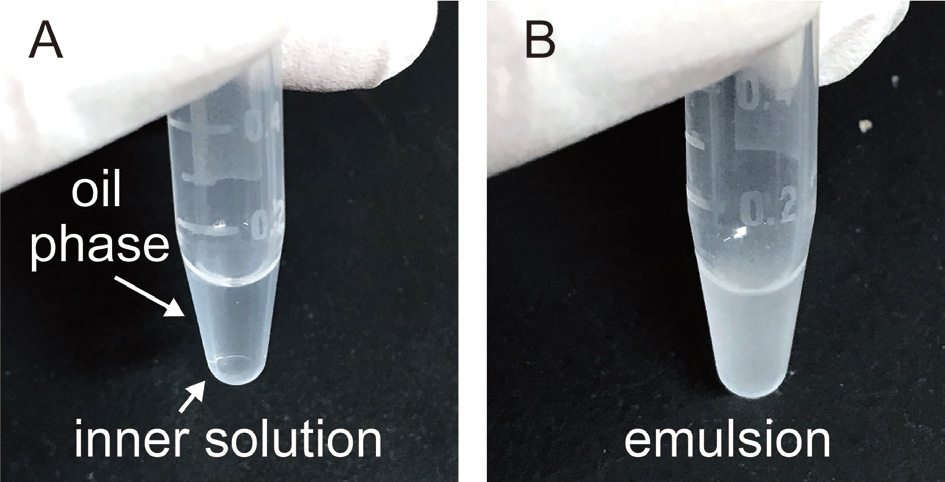
A representative result of emulsification. A. Before emulsification. B. After emulsification.
  3. Observation of self-organization of Min proteins inside the microdroplets
    - Prepare two cover glasses (we use 25 mm x 36 mm and 18 mm x 18 mm) to make a coverslip chamber by using a double-sided tape (about 90 µm thickness) as spacers (Figure 3).
    - Gently place a portion of the emulsion (15 μl) into the coverslip chamber (Note 10).
    - Observe self-organization of Min proteins inside the droplets by fluorescent microscope by 5-20 s intervals for 5 min or more at room temperature (ca. 25 °C). Generally, the exposure time was set to 500 ms for both GFP and mCherry. Generally, Min waves are very stable and continue at least 10 h. Thus, we can observe Min waves in many different (at least 10) positions with one coverslip chamber. A representative result is shown in Video 1.
      **Figure 3.**
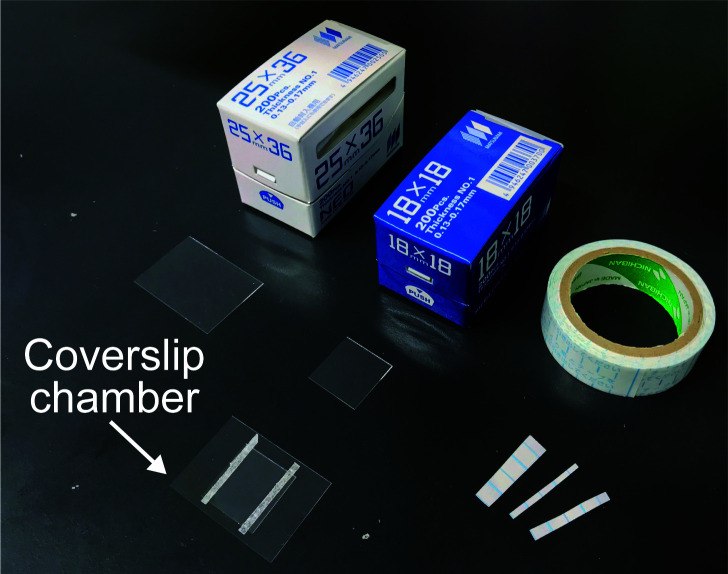
The coverslip chamber used for this protocol
      **Video 1.**
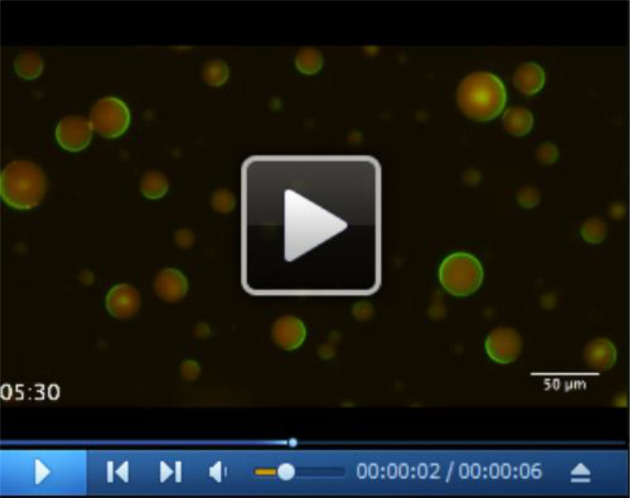
Wave propagation of Min proteins in lipid droplets. Time-lapse images of Min waves in lipid droplets emerged by this protocol. Green color indicates His-sfGFP-MinD and red color indicates MinE-mCherry-His. The link of the video is indicated at the end of the manuscript.

## Data analysis

- Image analysis
  1. Examination of Min wave emergence and its patterns
    - Open the tiff image file using Fiji software. Typically use 2,048 x 2,048 pixels, 16-bit, two-color channels for sfGFP-MinD and MinE-mCherry and time-lapse stack image.
    - Select “Image” “Adjust” and click the “Reset” button for every two-color channels (“Reset” button automatically adjusts the contrast between the minimal pixel intensity and maximum pixel intensity included in the object image).
    - If the contrast of the image is too low (usually high pixel intensity derived from protein aggregation cause this phenomena), manually adjust the contrast using “Adjust” submenu.
    - Run the time lapse movie and confirm whether Min protein waves appear or not [generally use at least 16 sequence images with 20 s intervals (total 5 min) to judge the wave appearance and wave patterns].
    - Classify the waves into 5 patterns by the behavior of His-sfGFP-MinD (traveling wave, standing wave, inhomogeneous membrane localization, homogenous membrane localization and homogeneous cytosol localization) as describes below.
      - Traveling wave: sfGFP-MinD continuously moves along the membrane of artificial cells. Typically traveling wave moves 1.5-2 rounds per 5 min under 1 µM His-sfGFP-MinD and 1 µM MinE-mCherry-His condition.
      - Standing wave: sfGFP-MinD alternately appears at each side of cell pole (axis of poles are stochastically chosen). Typically, indicates 2-3 rounds per 5 min under 1 µM sfGFP-MinD and 1 µM MinE-mCherry condition.
      - Inhomogeneous localization: sfGFP-MinD inhomogeneously localizes on membrane (typically occupies about half surface area of the inner membrane) but does not indicates dynamic movements within 5 min.
      - Homogeneous membrane localization: sfGFP-MinD homogeneously localizes on membrane and does not move.
      - Homogeneous cytosol localization: sfGFP-MinD homogeneously localizes inside droplets (cytosol) and does not move.
  2. Construction of kymograph
    - Convert the image to RGB color (select “Image” “Type” “RGB color”) or separate channels (select “Image” “Color” “Split Channels”) into 2 stack image files. To make composite image, the image should be separated into 2 colors and should be merged after making kymographs in every color.
    - Choose an artificial cell to construct kymograph.
    - Draw a circle along peripheral of the artificial cells selected using “Oval” tool (Figure 4A).
    - Select “Edit” “Selection” “Area to Line” to transform the circle to the curved line.
    - Select “Edit” “Selection” “Straighten”, input “5” into “Line Width” textbox, and check “Process Entire Stack” checkbox. Then click “OK” button to transform the curved line into straight line.
    - Select “Image” “Adjust” “Size” and uncheck “Constrain aspect ratio” check box. Then, input “1” to “Height” textbox, check “Average when downsizing” checkbox, and click “OK” button to compress the height of the image.
    - Select “Image” “Stacks” “Make Montage” and fill the numbers required with “1” for “columns”, the number of time points of the selected image for “Rows”, “1” for “Scale factor”, “1” for “First slice”, the number of time points of the selected image for “Last slice”, “1” for “Increment”, “0” for “Border width”.
    - Click “OK” button to create a kymograph image. Typical kymographs constructed from an image is shown in Figure 4B.
    - To merge two single-colored kymographs to one image, select “Image” “Color” “Merge Channels” and choose two images. Then click “OK” to make a composite image.
      **Figure 4.**
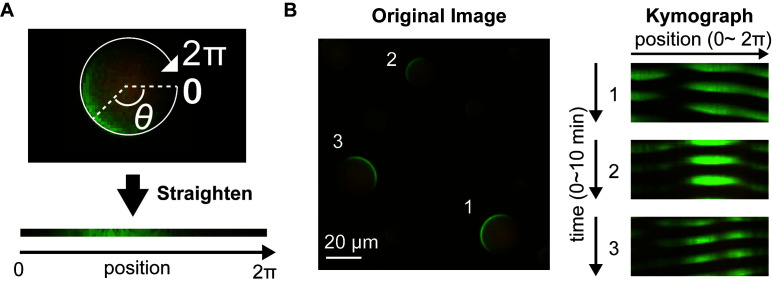
Kymograph construction of Min waves in artificial cells. A. A representative illustration of the straighten process over peripheral along artificial cells to construct kymographs. B. Examples of kymographs constructed from an image. The width of the kymograph was normalized by the 2π scale. The numbers of artificial cells indicated in the left image are corresponding to the kymograph numbers.

## Notes

1. Sequence information of His-sfGFP-minD and MinE-mCherry-His are shown in Supplementary document. We are ready to share the plasmids if the reader have interest use those.
2. RIPL strain may be replaced with other strains with (DE3) such as original BL21(DE3).
3. The most important point of this step is the complete disruption of cells by sonication. Experimenters who use another sonicator should use a typical condition to disrupt *E. coli* cells by the sonicator.
4. Flash freezing is not needed.
5. Because overexpression of MinE causes growth inhibition, the protocol is different from the case of His-sfGFP-MinD.
6. Tutorials of measurement of band intensities by Fiji software (ImageJ) are available online. For example, https://www.youtube.com/watch?v=t9k8HFL88kk, http://www.yorku.ca/yisheng/Internal/Protocols/ImageJ.pdf.
7. These steps are performed in a draft chamber.
8. Because homogeneous dried film is preferable, slow drying (30 s or more to complete drying) by gentle gas flow is better.
9. Argon gas can be replaced with other inactive gas like N_2_ gas.
10. We recommend fast transfer of the mixture to the glass slide slip as soon as possible, although the time does not matter as we tested.

## Recipes

1. LB medium1 g Bacto trypton
  0.5 g yeast extract
  10 g NaCl per 1 L
2. LB agar medium with an antibioticLB medium with 1.5 % agar and 100 μg/ml ampicillin or 25 μg/ml kanamycin
3. Sodium phosphate buffer (pH 7.6)
  - 300 mM NaH_2_PO_4_ and 300 mM Na_2_HPO_4_ are respectively prepared by dissolving them in Ultrapure water
  - Mix them to adjust pH to 7.6 (*ca.* 13:87 v/v ratio of NaH_2_PO_4_ and Na_2_HPO_4_)
4. HEPES-KOH (pH7.6)1 M HEPES is prepared, and pH is adjusted by KOH
5. Tris-HCl (pH 7.6)1 M Tris is prepared, and pH is adjusted by HCl
6. LS buffer50 mM NaH_2_PO_4_ (pH 7.6)
  300 mM NaCl
  10 mM imidazole
  1 mM dithiothreitol (DTT)
  0.1 mM phenylmethylsulfonyl fluoride (PMSF)
7. LS-ADP bufferLS buffer with 0.2 mM ADP
8. WS buffer50 mM NaH_2_PO_4_ (pH 7.6)
  300 mM NaCl
  20 mM imidazole
  10% glycerol
  0.1 mM EDTA
  0.1 mM PMSF
9. EL buffer50 mM NaH_2_PO_4_ (pH 7.6)
  300 mM NaCl
  250 mM imidazole
  10% glycerol
  0.1 mM EDTA
  0.1 mM PMSF
10. Storage buffer50 mM HEPES-KOH (pH 7.6)
  150 mM GluK
  10% glycerol
  0.1 mM EDTA
11. HG buffer50 mM HEPES-KOH (pH 7.6)
  10% glycerol
  0.1 mM EDTA
12. IEX-A buffer50 mM HEPES-KOH (pH 7.6)
  50 mM NaCl
  10% glycerol
  0.1 mM EDTA
13. IEX-B buffer50 mM HEPES-KOH (pH 7.6)
  1 M NaCl
  10% glycerol
  0.1 mM EDTA
14. CBB staining solution100 mg CBB, 5 ml ethanol, 13.7 g Citric acid, 5 g β-cyclodextrin per 1 L
15. RE buffer25 mM Tris-HCl (pH 7.6)
  150 mM GluK
  5 mM GluMg
