# Supplementary material for: Self-organization Assay for Min Proteins of Escherichia coli in Micro-droplets Covered with Lipids
Source: Bio Protoc. 2020 Mar 20;10(6):e3561. doi: 10.21769/BioProtoc.3561 (PMC7842281; doi:10.21769/BioProtoc.3561)
Supplement: Supplementary document [file BioProtoc-10-06-3561-s001.docx]

>His-sfGFP-MinD

ATGGGCAGCAGCCATCATCATCATCATCACAGCAGCGGCCTGGTGCCGCGCGGCAGCCAT

ATGAGTAAAGGAGAAGAACTTTTCACTGGAGTTGTCCCAATTCTTGTTGAATTAGATGGTGATGTTAATGGGCACAAATTTTCTGTCCGTGGAGAGGGTGAAGGTGATGCAACAAACGGAAAACTTACCCTTAAATTTATTTGCACTACTGGAAAACTACCTGTTCCATGGCCAACACTTGTCACTACTTTAACTTATGGTGTTCAATGCTTTTCCCGTTATCCGGATCACATGAAACGGCATGACTTTTTCAAGAGTGCCATGCCCGAAGGTTATGTACAGGAACGCACTATATCTTTCAAAGATGACGGGACCTACAAGACGCGTGCTGAAGTCAAGTTTGAAGGTGATACCCTTGTTAATCGTATCGAGTTAAAAGGTATTGATTTTAAAGAAGATGGAAACATTCTCGGACACAAACTCGAGTACAACTTTAACTCACACAATGTATACATCACGGCAGACAAACAAAAGAATGGAATCAAAGCTAACTTCAAAATTCGCCACAACGTTGAAGATGGATCCGTTCAACTAGCAGACCATTATCAACAAAATACTCCAATTGGCGATGGCCCTGTCCTTTTACCAGACAACCATTACCTGTCGACACAATCTGTCCTTTCGAAAGATCCCAACGAAAAGCGTGACCACATGGTCCTTCTTGAGTTTGTAACTGCTGCTGGGATTACACATGGCATGGATGAGCTCTACAAA GCACGCATTATTGTTGTTACTTCGGGCAAAGGGGGTGTTGGTAAGACAACCTCCAGCGCGGCCATCGCCACTGGTTTGGCCCAGAAGGGAAAGAAAACTGTCGTGATAGATTTTGATATCGGCCTGCGTAATCTCGACCTGATTATGGGTTGTGAACGCCGGGTCGTTTACGATTTCGTCAACGTCATTCAGGGCGATGCAACGCTAAATCAGGCGTTAATTAAAGATAAGCGTACTGAAAATCTCTATATTCTGCCGGCATCGCAAACACGCGATAAAGATGCCCTCACCCGTGAAGGGGTCGCCAAAGTTCTTGATGATCTGAAAGCGATGGATTTTGAATTTATCGTTTGTGACTCCCCGGCAGGGATTGAAACCGGTGCGTTAATGGCACTCTATTTTGCAGACGAAGCCATTATTACCACCAACCCGGAAGTCTCCTCAGTACGCGACTCTGACCGTATTTTAGGCATTCTGGCGTCGAAATCACGCCGCGCAGAAAATGGCGAAGAGCCTATTAAAGAGCACCTGCTGTTAACGCGCTATAACCCAGGCCGCGTAAGCAGAGGTGACATGCTGAGCATGGAAGATGTGCTGGAGATCCTGCGCATCAAACTCGTCGGCGTGATCCCAGAGGATCAATCAGTATTGCGCGCCTCTAACCAGGGTGAACCGGTCATTCTCGACATTAACGCCGATGCGGGTAAAGCCTACGCAGATACCGTAGAACGTCTGTTGGGAGAAGAACGTCCTTTCCGCTTCATTGAAGAAGAGAAGAAAGGCTTCCTCAAACGCTTGTTCGGAGGATAA

>MinE-mCherry-His

ATGGCATTACTCGATTTCTTTCTCTCGCGGAAGAAAAACACAGCCAACATTGCAAAAGAACGGCTGCAGATTATTGTTGCTGAACGCCGTCGCAGCGATGCAGAACCGCATTATCTGCCGCAGTTGCGTAAAGATATTCTTGAGGTCATTTGTAAATACGTACAAATTGATCCTGAGATGGTAACCGTACAGCTTGAGCAAAAAGATGGCGATATTTCTATTCTTGAGCTGAACGTGACCTTACCGGAAGCAGAAGAGCTGAAA

GTGAGTAAAGGCGAGGAGGACAATATGGCGATCATCAAAGAGTTCATGCGCTTCAAAGTCCACATGGAAGGCAGCGTTAATGGTCACGAGTTCGAAATTGAGGGCGAAGGCGAAGGTCGTCCGTATGAGGGTACACAGACCGCTAAACTGAAAGTCACGAAAGGTGGTCCACTGCCATTTGCTTGGGATATTCTGAGCCCACAGTTCATGTATGGCTCCAAAGCCTATGTGAAACATCCGGCCGATATTCCGGACTATCTGAAACTGAGCTTCCCTGAAGGGTTCAAATGGGAACGTGTGATGAACTTTGAGGATGGTGGTGTTGTGACAGTGACACAGGATTCTAGCCTGCAAGACGGTGAGTTCATCTATAAAGTGAAACTGCGTGGCACGAATTTTCCGAGTGATGGCCCGGTTATGCAGAAAAAAACGATGGGTTGGGAGGCCTCTAGTGAGCGTATGTATCCAGAAGATGGCGCTCTGAAAGGCGAAATCAAACAGCGTCTGAAACTGAAAGATGGTGGCCACTATGATGCCGAAGTGAAAACCACGTATAAAGCCAAAAAACCTGTCCAACTGCCTGGTGCCTATAACGTTAACATCAAACTGGACATCACCTCACACAATGAGGACTATACGATCGTGGAGCAGTATGAGCGTGCTGAAGGACGTCATTCTACCGGTGGTATGGATGAGCTGTATAAA

GGATCCCTCGAGCACCACCACCACCACCACTGA
